# Application of machine learning in bacteriophage research

**DOI:** 10.1186/s12866-021-02256-5

**Published:** 2021-06-26

**Authors:** Yousef Nami, Nazila Imeni, Bahman Panahi

**Affiliations:** 1 Department of Food Biotechnology, Branch for Northwest & West Region, Agricultural Biotechnology Research Institute of Iran, Agricultural Research, Education and Extension Organization (AREEO), Tabriz, Iran; 2 Young Researchers and Elite Clube, Marand Branch, Islamic Azad University, Marand, Iran; 3 Department of Genomics, Branch for Northwest & West Region, Agricultural Biotechnology Research Institute of Iran, Agricultural Research, Education and Extension Organization (AREEO), Tabriz, Iran

**Keywords:** Machine learning, Bacteriophage, Classification, Host, Life cycle

## Abstract

Phages are one of the key components in the structure, dynamics, and interactions of microbial communities in different bins. It has a clear impact on human health and the food industry. Bacteriophage characterization using in vitro approaches are time/cost consuming and laborious tasks. On the other hand, with the advent of new high-throughput sequencing technology, the development of a powerful computational framework to characterize the newly identified bacteriophages is inevitable for future research. Machine learning includes powerful techniques that enable the analysis of complex datasets for knowledge discovery and pattern recognition. In this study, we have conducted a comprehensive review of machine learning methods application using different types of features were applied in various aspects of bacteriophage research including, automated curation, identification, classification, host species recognition, virion protein identification, and life cycle prediction. Moreover, potential limitations and advantages of the developed frameworks were discussed.

## Background

Bacteriophages (phage) are prokaryotic viruses that infect and replicate into the bacteria with the host specificity manner. Among the well-characterized phages, the vast majority have a single genome [18]. It has been estimated that nearly 85% of phages are double-stranded (ds) DNA enveloped by a protein shell [1]. Hence, it has been proposed that phages with ds DNA genome are amongst the most plentiful entities on Earth [13].

Phages infect the particular bacterial hosts and highjack the host-cell machinery in the lytic (or virulent) life-style for replicating as well as destroying the host. Therefore, they concurrently produce progeny and kill the hosts. Nevertheless, the existence of diverse phages in nature, provides a valuable resource as antibacterial agents and infection control. Moreover, researchers utilized phage typing to identify the subtypes and species of bacteria. In addition, they are prominent drivers of biogeochemical cycles on Earth [36] and the major actors in leading and raising bacterial diversity [7].

Two culture-based and in silico approaches are used for studying the bacteriophages. The culture-based approach is costly and laborious, especially in high-throughput sequencing experiments. To resolve this problem, the use of insilico approaches such as intelligent data mining and knowledge discovery are among the most promising alternative [32].

**Fig. 1:**
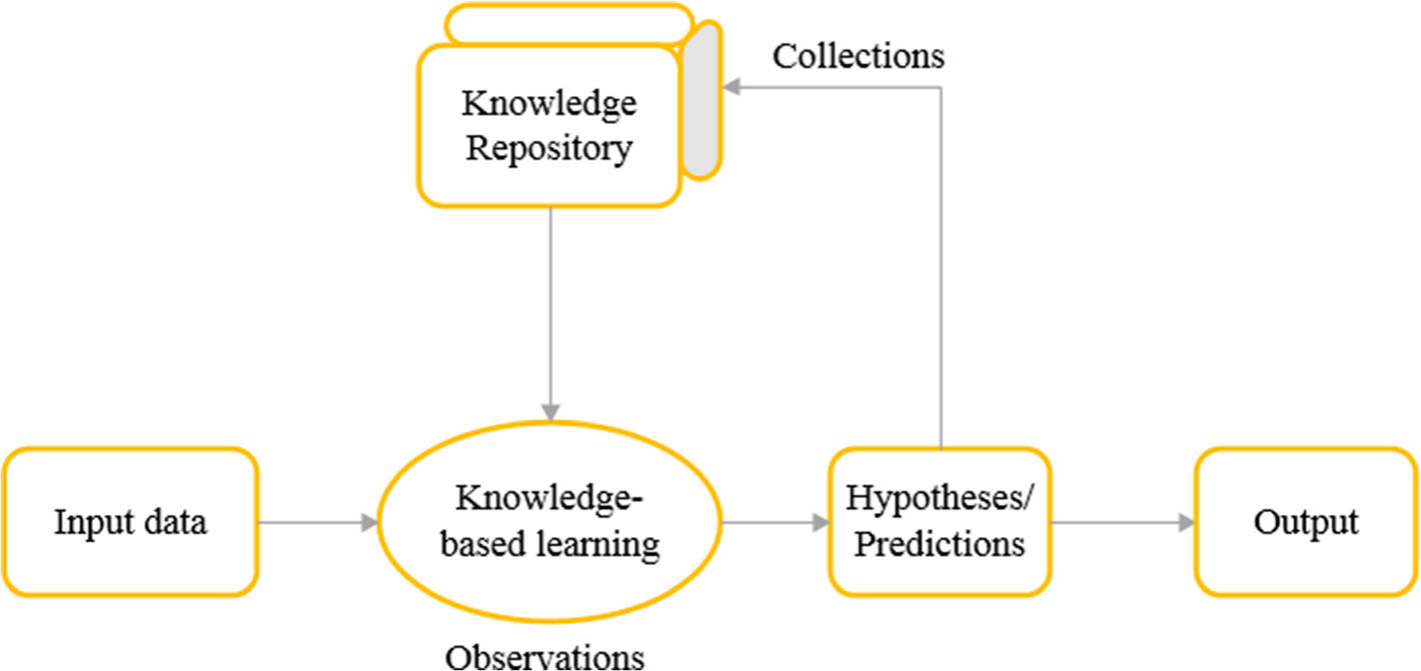
Machine learning process schedule

Among different data mining methods, machine learning techniques have gained considerable prominence in bacteriophage researches. Machine learning (ML) refers to knowledge and pattern discovery in empirical data using statistical, probabilistic, and optimization methods. Machine learning procedures classified into three modeling strategies: unsupervised, supervised, and semi-supervised learning models. Unsupervised learning is a type of machine learning algorithm used to draw inferences from unknown datasets that is neither classified nor labeled [28, 30]. Some examples of unsupervised learning algorithms are K-means and K-nearest neighbors (KNN) for clustering problems and Apriori algorithm for association rule learning problems. Supervised learning algorithms try to make predictions based on evidence (labeled data) in the presence of uncertainty [16, 29]. Support vector machine (SVM), Decision tree (DT), Random Forest (RF), and Naïve Bayes (NB) are some popular examples of supervised algorithms. In semi-supervised learning methods, which conceptually situated between supervised and unsupervised learning, a small amount of labeled data with a large amount of unlabeled data are combined to perform certain learning tasks [31]. Overall steps in the implementation of any machine learning models are presented in Fig. 1. Choosing the machine learning algorithm is dependent upon the learning strategy as well as the analysis goal.

In the present study we comprehensively reviewed the possible use of ML in several dimensions of bacteriophage research, in particular, prediction, classification, host prediction, recognition of the life-cycle, and characterization of invasive proteins.

## Automated recovery, prediction and classification of bacteriophage

Phages infecting bacteria have been proposed as the main determinant in dynamics, interaction, and structures of the microbial communities [19]. With the development of next-generation high-throughput sequencing, computational identification of virus genomes from different bins is a critical step [32]. Metagenomics is a well-fitted approach to provide insights into the composition, structure, and dynamics of environmental viral communities. At present, gene-based similarity methods are popularly used to recovery, annotation, and curation bacteriophages from mixed metagenomic assemblies; however, these techniques have low performance due to higher diversity and less information of gene content and genomic structures of bacteriophages. Therefore, some computational tools based on ML algorithms have been developed to improve the automated recovery and prediction of bacteriophages. MARVEL (Metagenomic Analysis and Retrieval of Viral Elements) as a ML based tools was developed to predict double-stranded DNA bacteriophage sequences in metagenomic bins. MARVEL leverage the information of annotation and sequence signature from previosly identified phages for identifying the double-stranded phages in metagenomic bins [5]. In this tool, six genomic features including the average gene length, the average spacing between genes, the density of genes, frequency of strand shifts between neighboring genes, ATG relative frequency, and fraction of genes with significant hits against the pVOGs database were extracted from the baseline dataset of RefSeq. Then, training performs based on the random forests model [5].

VirFinder is another tool that was developed for virus overlap group identification without the need to sequence signature and the annotation databases [33]. For prediction of phages, VirFinder employs the *k*-mer features of query sequences and then generates a score between 0 and 1 based on the logistic regression model with lasso regularization as a trained machine learning model [33]. It has been demonstrated that VirFinder can be periodically updated by training it on new and available sequences [33].

Another tool was developed by [37] to identify the viral signals based on both reference dependence and independence manner. VirSorter is largely reliant on the database searches of the anticipated proteins with the use of probabilistic similarity and reference homology for compiling metrics of the virus-like proteins enrichment and concurrent depletion of other proteins [37]. Hence, it utilizes a virus-specific curated database Pfam [15] for the nonvirus annotations, although it would not completely differentiate viral from the nonviral Pfam annotations. According to the study by [5], MARVEL, VirFinder, and VirSorter have a comparable performance on specificity, but MARVEL has a better recall (sensitivity) performance [5].

Kieft et al. [22] also developed VIBRABT for automated recovery, annotation and curation of microbial viruses, and evaluation of viral community function from genomic sequences. VIBRANT was the first method to utilize neural networks and protein similarity approach. Based on the author’s declaration, VIBRANT recovered an average of 94% of the viruses with higher performance than VirFinder, VirSorter, and MARVEL.

By increasing the number of identified uncertain phage genomes [39], the development of a flexible and integrative tool for fast and precise taxonomic description is inevitable. Conventional approaches to tackle the phage categorization have been established based on virion morphology characterization with the Transmission Electron Microscopy (TEM) and sequence-based strategies [35]; however, they occasionally dealt with the genus and characterization have been done at family and subfamily level [38]. Additionally, the classification of the phages based on the experimental data is a time and labor-consuming procedure. Machine learning has been considered an attractive alternative for classifying the bacteriophages [20]. Successful utilization of ML methods in the classification of a bacteriophage using ANN has been reported by [10]. They developed a novel integrative tool for classifying phages called ClassiPhage 2.0. In ClassiPhage 2.0, the authors have made Hidden Markov Models (HMMs) profiles by scanning the available phage proteomes. Created phage-derived-HMMs scoring matrix was used to train a model using ANN algorithm to classify phage genomes into 12 phage family [10]. According to the authors’ declaration, their proposed model could also be extended to include more features than HMM profile hits. However, there are two limitations with ClassiPhage 2.0. First, as genes or proteins can be shared by different taxa, alignment may lead to ambiguous label assignment. Second, given the enoumerous diversity of the phages, alignment-based methods are not able to assign taxa for new species with novel proteins or lack quality alignments with the references. Thus, using only sequence similarity cannot provide ideal resolution.

Another ML-based tool to phage classification is vConTACT 2.0 which utilizes a clustering algorithm to leverage gene organization conservation for phage classification [21]. If the reference genomes and contigs are in the same cluster, the labels of the reference genomes will be assigned to those contigs. While vConTACT 2.0 present satisfactory performance on classification of complete genomes, the classification accuracy decreases in the contigs with short length. It has been demonstrated that shorter contigs do not contain many proteins and thus do not lead to valid edges in the gene-sharing network [41]. Therefore, the clustering algorithms fail to group contigs and reference genomes in the same cluster.

To deal with the above-mentioned issues in the bacteriophage calassification, PhaGCN was developed recently [41]. PhaGCN utilizes the semi-supervised learning framework that knowledge graph is constructed by combining the DNA sequence features learned by convolutional neural network (CNN) and protein sequence similarity gained from gene-sharing network. Then graph convolutional network (GCN) was applied to utilize both the labeled and unlabeled samples in training to enhance the learning ability. The major improvement of PhaGCN stems from combined strength of the reference-based model and the learning-based model using the knowledge graph: the nodes contain automatically learned features from nucleotide sequences and the edges are created by protein-based alignment.

Classification performance of PhaGCN, vConTACT, and ClassiPhage were compared using simulated and real sequencing data. The result shows that PhaGCN always has the largest number of predicted contigs with the highest classification accuracy. Moreover, in contrast to the vConTACT and ClassiPhage, the classification performance of PhaGCN is stable with the change of the contig length, making it useful for classifying short contigs [41].

## Phage host prediction

Determining the host species of identified phages is an important challenge in virology. Owing to the recent development of sequencing technology an increased amount of newly identified viruses have been discovered within different ecological niches. However, the host species of the majority of these viruses remains unknown. To address this gap, culture-based and computational methods were reported. In culture-based procedures, the host ranges of phage are identified by the growth of the host bacteria with phages over an agar plate [47]. Since viromes revealed enormous diversity of viruses with no isolated relatives, linking these viruses with their putative hosts by culture-independent methods has become important to gain insights into the ecology of viruses. Computational approaches to virus-host prediction fall into four strategies: searching for homologous sub-sequences in the hosts [14, 38]; looking for co-abundance between virus and host [3]; distance-based metrics of oligo-nucleotide or k-mer composition [3]; and machine learning methods [45]. Most of the mentioned approaches rely on reference genomes, and the availability of the genomes is a major drawback. However, machine learning approaches relying on training dataset that are independent of reference genomes or alignment steps [49]. A majority of the machine-learning strategies for predicting the virus-host employed characteristics extracted from nucleotide similarity-based k-mer biases such as CpG bias, di-codon bias, and CG bias [27]. Aguas and Ferguson [2] successfuly applied nucleotide or amino acid features to predict host species using RF-based models in RNA viruses. In another study, hosts of viruses were successfully predicted using a dual discriminants model including SVM and Mahalanobis distance (MD) [43]. Moreover, successful application of logistic regression, support vector machines, random forest, Gaussian naive Bayes, and Bernoulli naive Bayes to predict phage’s host on genus level using oligonucleotide frequencies was reported [52]. One of the most important characteristics of this tool is a plant-, vertebrate-, and arthropod-specific virus’s identification ability. However, poor efficacy was observed in the arthropod-specific viruses host prediction.

**Fig. 2:**
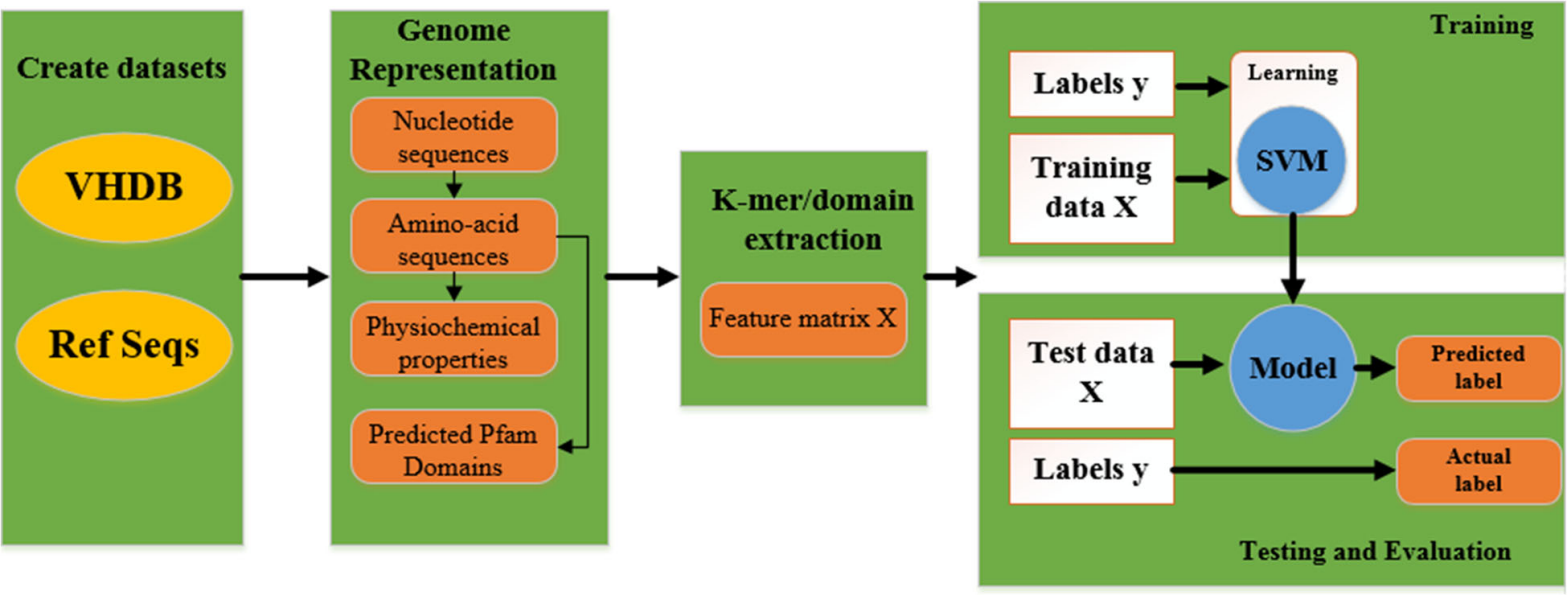
Workflow for extracting and selection of genome features and subsequently bacteriophage hosts prediction using SVM classifiers [49]

All of the above-mentioned methods have been used the information of the nucleotide sequence for a virus hosts prediction. Whereas, information of amino acid sequences and their biochemical properties and subsequent functional properties have not been considered. Although all of the ‘functional’ information is present in the nucleotide sequence, it is not necessarily in a form that is easy for machine learning approaches to extract. Based on this hypothesis, Young et al. [49] developed a new computational framework for host prediction by combining k-mer compositions and protein domains of bacteriophage genomes [49]. They trained and tested SVM classifiers to compare the predictive capacity of each of the genome representations including nucleic acid sequence k-mers, amino acid sequence k-mers, physiochemical properties of the amino acid sequence, and predicted Pfam domain as depicted in Fig. 2.

Their results showed that the host prediction accuracy improved with increasing k-mer length for all k-mer based features [49]. However, methods based on K-mers in general have decent prediction accuracy, though the mechanisms behind this phenomenon is not fully understood. More recently, Wang et al. [46] developed a tool known VirHostMatcher-Net using a network-based integrated framework to predict bacteriophages hosts. In this tool, CRISPR sequences, alignment-free similarity, and co-abundance were applied as features to train the model using SVM and RF methods [46]. The authors showed that the VirHostMatcher-Net, comparison with other developed tools, improves the host prediction rate up to 6-fold. It seems that the frameworks with the combining of multi-layers information improve the modeling and prediction performance.

## Bacteriophage virion proteins prediction

Binding of phage virion proteins (PVP) to the host surface is a critical step for the injection of genetic materials into bacteria during the infection procedure of phages. Identification of PVPs such as endolysins, exopolysaccharides, and holins is important for deciphering the complex dynamics of connection between phage and the host bacteria to develop effective antibacterial drugs or antibiotics [26]. It has been shown that the identification of PVPs using experimental procedures including mass spectrometry, sodium dodecyl sulfate-polyacrylamide gel electrophoresis, and protein arrays is laborious and expensive [34]. Additionally, sequence-based computational approaches are needed before the in vitro experiments [40]. Although, limited experimented data is a major drawback for traditional in silico approaches, Machine learning approaches provided a promising avenue to predict the functions of phage proteins [26, 50, 53]. Summary of ML-based identifiers for prediction of PVPs are presented in Table 1.

**Table 1:** Summary of ML models and features were used for training PVPs

No.	Predictor	Method	Number dataset (TR/TS)	Performance
1	ANN	“ACC, protein isoelectric Points” + ANN	307 (307/NA)	85%
2	Naïve Bayes	“ACC, DPC” + CFS + Naïve Bayes	401 (307/94)	79%
3	PVPred	g-gap DPC + ANOVA+SVM	307 (307/NA)	85%
4	PhagePred	g-gap DPC + ANOVA + Multinomial Naïve Bayes	307 (307/NA)	98%
5	PVP-SVM	“AAC, ATC, CTD, DPC, PCP” + RF-based feature selection + SVM	401 (307/94)	87%
6	SVM-based	g-gap DPC + “ANOVA, mRMR” + SVM	401 (307/94)	86%
7	Ensemble RF	“CTD, bi-profile Bayes, PseAAC, PSSM” + Relief + RF	501 (253/248)	85%
8	Pred-BVP-Unb	CT, SAAC, bi-PSSM+SVM	401 (307/94)	92%
9	PVPred-SCM	DPC + SCM	401 (307/94)	77%
10	Meta-iPVP	Probabilistic feature+SVM	626 (313/313)	82%

*SCM* scoring card method, *SVM* support vector machine, *AAC* amino acid composition, *ATC* atomic composition, *bi-PSSM* bi-profile position specific scoring matrix, *CTD* chain-transition-distribution, *CT* composition and translation, *DPC* dipeptide composition, *GDPC* g-gap dipeptide composition, *PCP* physicochemical properties, *SAAC* split amino acid composition, *TR* training dataset, *TS* testing dataset

In this regard, Seguritan et al., [40] developed an ANN model, using amino acid composition (AAC) and protein isoelectric points as input features for the classification of viral structural proteins. In another study, Feng et al. [17] employed the amino acid and dipeptide composition features for model development using a naïve Bayesian algorithm. Ding et al. [12] also developed a SVM-based prediction tool known as PVPred. In this tool, the researchers utilized a one-gap dipeptide occurrence frequency as a feature for the model training using the SVM algorithm. Another study dealt with the SVM discriminant to study PVPs using the chosen optimal g-gap dipeptide composition as a feature [42]. Furthermore, in another study, a publicly available method was developed using a RF-based ensemble method to identify PVPs [51]. To improve the accuracy and transferability of the prediction model that was developed by Zhang et al. [51], a SVM-based PVP predictor called PVP-SVM was developed by Manavalan et al., [24]. In PVP-SVM predictor, RF and extremely randomized tree (ERT)-based models were applied for prediction of PVPs using AAC, atomic and dipeptide composition, and chain-transition-distribution features [24]. Each strategy considered a machine learning as one of the optimal computational methods, which is affordable, simplified, efficacious as well as reproducible in comparison with the conventional experimentations [48]. Recently, a machine learning model-based tool, known Pred-BVP-Unb was developed with other features, i.e. Bi-PSSM evolutionary information, composition & translation, and split amino acid composition [6]. In the Pred-BVP-Unb, attribute selection is perfomed by a recursive feature elimination algorithm. Then, selected features is used to train a model by SVM using radial base kernel [6]. It has been declared that the mentioned tool can predict PVPs with 92.54% for the benchmark dataset [6]. In other study, different machine learning algorithm efficiency to predict PVPs using wide range features were comparatively surveyd and shown the g-gap DPC (dipeptide composition) is the most essential feature for predicting of PVPs. They compared the prediction accuracy of SVM, NB, RF, and ensemble methods, of which SVM was the more effective discriminator [26]. However, the problem was far from being solved. First, low prediction accuracy due to poor protein motifs representation. Secondly, the class imbalance leads to classification errors and biasness problem. Thirdly, a robust feature selection algorithm is required to rank and select the best discrimination feature subset for the model prediction. Moreover, they are not generalized or transferable to researchers with informatics background who can develop in-house prediction models [8, 9]. Motivated by the above mentioned limitations, Meta-iPVP were proposed which was employed the eficient feature representation approach to generate discriminative probabilistic features using SVM algorithm [9]. Performance evaluation showed that the Meta-iPVP could distinguish PVPs and non-PVPs with 0.817 and 0.642 accuracy and MCC, respectively, which corresponds to 6–10% and 14–21% improvements over above-mentioned predictors [9]. Other algorithm has proposed PVPred-SCM and VirionFinder. In PVPred-SCM, the propensities of dipeptides to be PVPs were calculated. Then, the propensity scores of all dipeptides were optimized using genetic algorithm. Results showed that PVPred-SCM had higher performance, compared to SVM-based tools with various types of protein features [8]. Regarding the VirionFinder, this predictor considers the protein fragments rather than complete proteins as training data sets, which helps to extract more information and consequently predict PVPs more effectively than previous methods.

## Recognition of the bacteriophage life-cycle using ML

Virulent phages which have a largely lytic lifecycle following bind to the host cell, and inject the nucleic acid material for using the bacterial replication and translation machinery. Subsequently, the bacterium would be lysed and bacteriophages would be released in the environment. On the contrary, the lysogenic cycle which is followed by temperate phages, phage genome is embedded into a bacterial genome to forms a prophage; a situation where it may take several generations. However, in stress conditions, prophages may be shifting their life cycle to lytic mode [23]. Bacteriophage life cycle knowledge i.e. population dynamics, and virulent lifestyle are employed for phage therapy as a bio-control strategies [4]. Commonly, phage lifestyle is specified by in vitro culturing, isolation and characterization. With the emergence and the development of new sequencing platforms and identification of novel phages in different bins, the development of rapid computational approaches for the determination of lifestyle is inevitable [25]. In the early effort, phage lifestyle has been determined using the tetra-nucleotide frequency in phage and respective hosts [11]. Nevertheless, the availability of the host genome is a major drawback in this regard [25]. Machine learning-based methods are proposed as a promising approach for resolving this drawback with the prediction of lifestyle using phage nucleotide sequences [44]. In earlier study, Phage Classification Tool Set (PHACTS) was developed to predict the phage life style using the amino-acid sequence characteristics as features [25]. Then, the created a similarity matrix was trained using RF classifier to predict that the phage is lytic or lysogenic. Although the PHACTS are shown to have a 99% precision rate, however, due to imperfect annotation of newly identified phages it is not possible to make a confident life style prediction using the PHACTS [25]. To resolve this problem, a machine learning-based tool known as PhageAI was recently developed by Tynecki et al. [44] which requires just a DNA nucleotide sequence of phages to predict that phage life style is lytic or lysogenic. In the PhageAI, feature selection is performed using ranking are performed using recursive feature elimination, and cross-validated selection of the best number of features (RFECV). Then, models are trained using supervised ML algorithms including Multinomial NB, SVM, Support Vector Classifier (SVC), SGD, Logistic Regression, multilayer perceptron (MLP), RF, K Neighbors, Gradient Boosting, XGBoost, CatBoost, Light GBMc Classifiers. PhageAI are shown to have about 99% prediction accuracy based on SVM algorithm [44].

## Conclusion

Our review summarizes and discusses the use of machine learning in the analysis of various aspects of the bacteriophage field. The majority of studies carried out in this field thus far have demonstrated promising results. According to the comparisons of all discriminators, we concluded that the utilization of different feature descriptors which harbors the multi-layer information is critical for training datasets enrichment. Moreover, in most of the studies, different features have been linearly combined and ranking of the feature were performed. However, the employment of feature descriptors fusion may improve the prediction accuracy of models. Additionally, the application and optimization of deep learning techniques can improve our knowledge about bacteriophage characteristics.

## Data Availability

Not applicable.

## Abbreviations

ACC: Amino Acid Composition
CNN: Convolutional Neural Network
DT: Decision Tree
DPC: Dipeptide Composition
ERT: Extremely Randomized Tree
GCN: Graph Convolutional Network
HMMs: Hidden Markov Models
KNN: K-Nearest Neighbors
MD: Mahalanobis Distance
ML: Machine Learning
MARVEL: Metagenomic Analysis and Retrieval of Viral Elements
MLP: Multilayer Perceptron
NB: Naïve Bayes
PVP: Phage Virion Proteins
PHACTS: Phage Classification Tool Set
RF: Randome Forest
SVM: Support Vector Machine
SVC: Support Vector Classifier
TEM: Transmission Electron Microscopy
